# A Rare Outcome From a Self-Inflicted Gunshot Wound to the Neck

**DOI:** 10.7759/cureus.18063

**Published:** 2021-09-17

**Authors:** Raina Kishan, Saleena Ramzanali, Muhammad Nazim

**Affiliations:** 1 Surgery, Texas Tech University Health Sciences Center, Amarillo, USA

**Keywords:** gunshot injuries, cervical spinal injury, emergency and trauma radiology, mri in spinal trauma, imaging and trauma

## Abstract

The incidence of self-inflicted gunshot wounds has increased significantly in the civilian population. In this case, we present a 25-year-old male with a self-inflicted gunshot wound to the neck exiting to the left shoulder. Penetrating injuries to the neck carry a high likelihood of severe injury and death. Exsanguination due to damage to the carotid or vertebral arteries is the most common cause of immediate death. Traumas caused by gunshot wounds can be complicated by an unusual path and can be devastating, depending on the extent of the injury. Our patient presents with a unique singular outcome of a spinal cord injury from anterior penetrating neck trauma. The aim of this case report is to raise awareness of a unique outcome from a self-inflicted gunshot wound, as it is vital to be aware of all possible outcomes because these injuries become more prevalent in our communities.

## Introduction

The incidence of self-inflicted gunshot wounds has increased significantly in the civilian population. The total number of injuries from gun violence (willful, malicious, or accidental) in 2019 was 30,186 and was noted to have a 30% increase in 2020 to a total of 39,492 [1]. In this case, we present a 25-year-old male with a self-inflicted gunshot wound to the neck exiting to the left shoulder. Penetrating injuries to the neck carry a high likelihood of severe injury and death. Exsanguination due to damage to the carotid or vertebral arteries is the most common cause of immediate death [2]. Penetrating neck injuries are categorized by their anatomical zone. Zone 1, the most caudal of the regions, is the area from the sternal notch to the cricoid cartilage and contains many important structures, including the subclavian vessels, internal jugular veins, proximal carotid artery, vertebral artery, trachea, esophagus, thoracic duct, and the apices of the lungs [3]. Zone 2 is the area from the cricoid cartilage to the angle of the mandible and contains the common carotid arteries, vertebral arteries, jugular veins, trachea, esophagus, larynx, and pharynx [3]. Finally, Zone 3 extends from the angle of the mandible to the base of the skull and contains the distal portions of the internal carotid arteries, vertebral arteries, jugular veins, and pharynx [3]. Mortality from a penetrating neck injury appears to be highest in Zone 1 due to its proximity to the vital organs in the mediastinum and the surgical difficulties that impose [4]. Other things to consider include secondary blast injuries, which are characterized by penetrating trauma that is associated with the explosive movement of materials related to gunshots. The aim of this case report is to describe a unique outcome of a self-inflicted gunshot wound and to raise awareness of these devastating incidents.

## Case presentation

**Figure 1:**
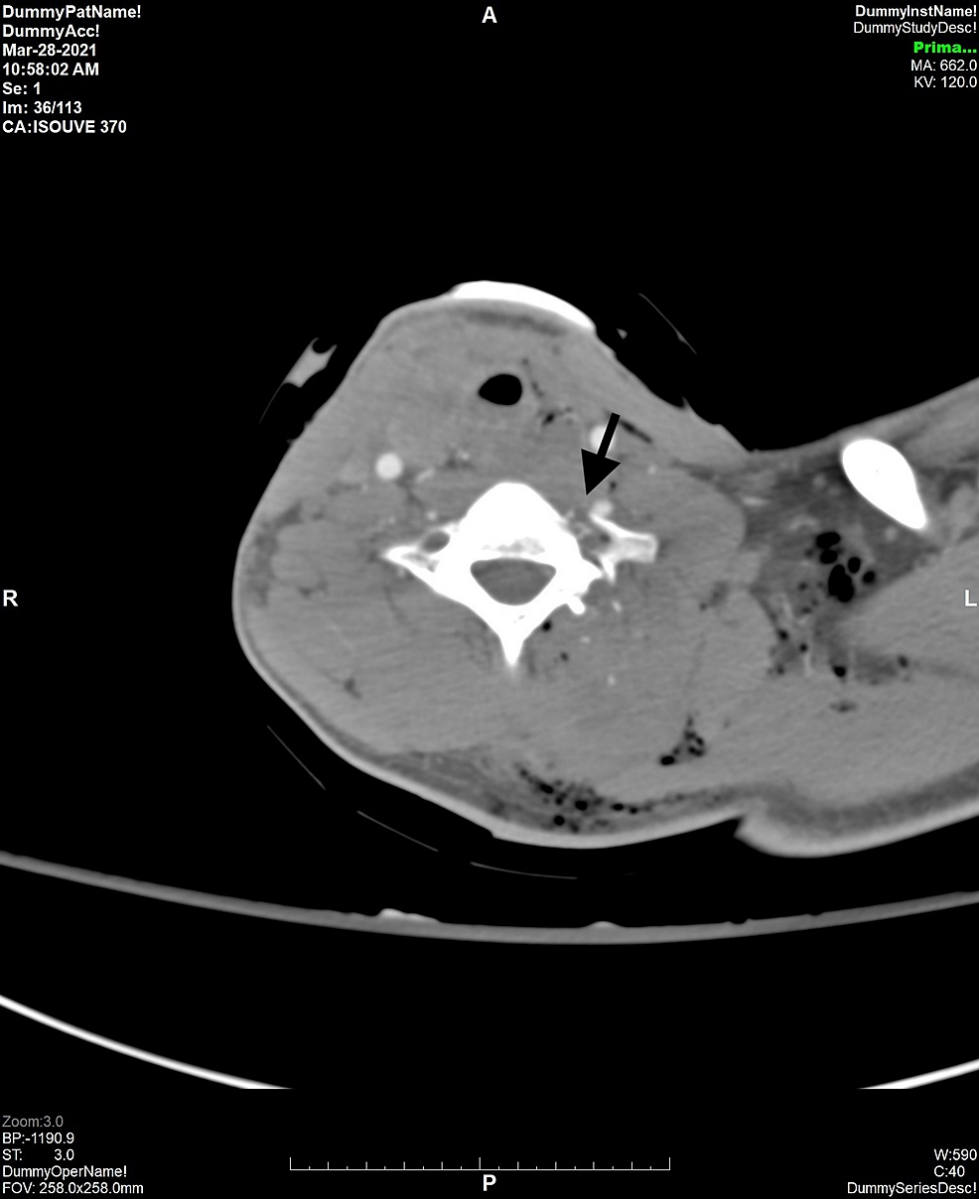
CT Image of the Cervical Spine (Axial View) Showing Fracture of the Left C7 Transverse Process (arrow)

**Figure 2:**
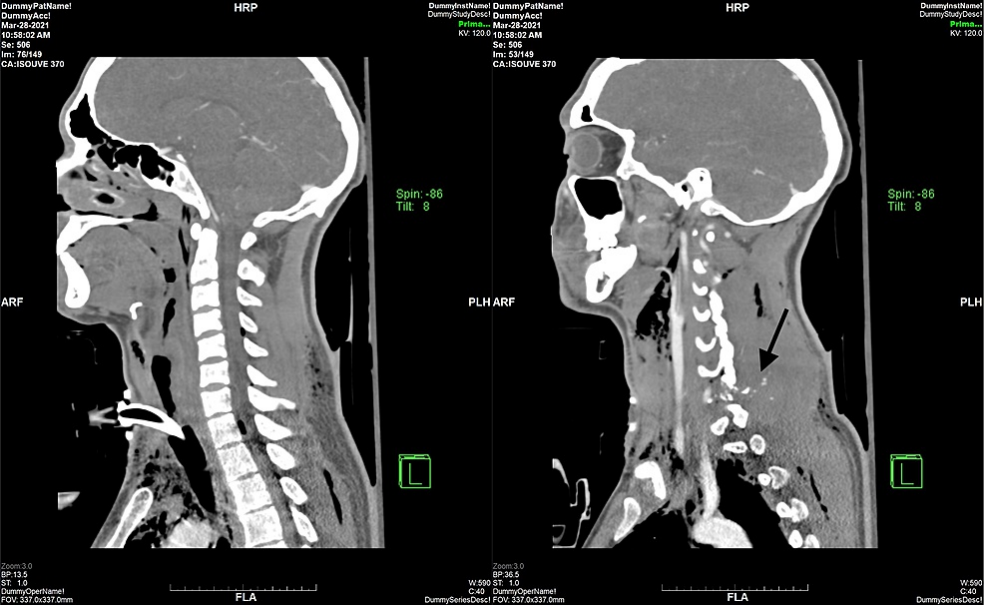
CT Images of the Cervical Spine (Sagittal view) Showing Tracheostomy Site and Multiple Comminuted Fracture Fragments (arrow)

A 25-year-old Caucasian male presented to the Northwest Texas Hospital emergency room (ER) for a self-inflicted gunshot wound to the midline of the neck, exiting to the left shoulder. The patient reported that he was cleaning his AR-15 rifle before accidentally shooting himself. Emergency medical services (EMS) were called to the site and reported bleeding and likely tracheal injury found in Zone 2. Multiple attempts to perform an emergent cricothyroidotomy by EMS were unsuccessfully performed and oxygen was delivered via an endotracheal tube (ET) tube through the gunshot wound into the trachea. Upon arrival to the ER, the patient was hypotensive at 62/29 and hypoxic with oxygen saturation (SpO2) of 75%. Massive transfusion protocol was initiated, and the patient was resuscitated. Orotracheal intubation was attempted in the ER but was unsuccessful, as the tube exited through the wound in the proximal trachea, indicating a likely full-thickness anterior tracheal ring injury. General surgery placed a #6 Shiley tracheostomy through the wound and attached it to a ventilator at that time, due to an inability to be intubated without the tube exiting the wound itself. The patient proceeded to achieve good oxygenation status. A fiberoptic bronchoscope through the tracheostomy confirmed proper placement and no major airway injury to the secondary and tertiary bronchioles. CT of the cervical spine showed evidence of an acute comminuted fracture of the left C7 transverse process, left lateral mass, left vertebral foramen, left lamina, and left superior and inferior facets (Figure 1), with multiple comminuted fracture fragments in the left C7-T1 lateral recess and left neural foramen (Figure 2).

CT angiography showed no damage to the aortic arch, carotid arteries, or vertebral arteries. A left-sided hemo/pneumothorax was found and a chest tube was placed by the ER physician. An esophagogastroduodenoscopy was performed, which showed no trauma to the esophagus. Initial CT scans showed intact cricoid and thyroid cartilages and bronchoscopy and laryngoscopy performed by thoracic surgery showed no evidence of damage to the trachea and proper vocal cord movement. The lack of damage to the inner structures suggests a unique and superficial trajectory of the bullet, in which the bullet entered the patient at the anterior neck, at the site of the tracheostomy placement, and exited the left posterior neck toward the shoulder.

**Figure 3:**
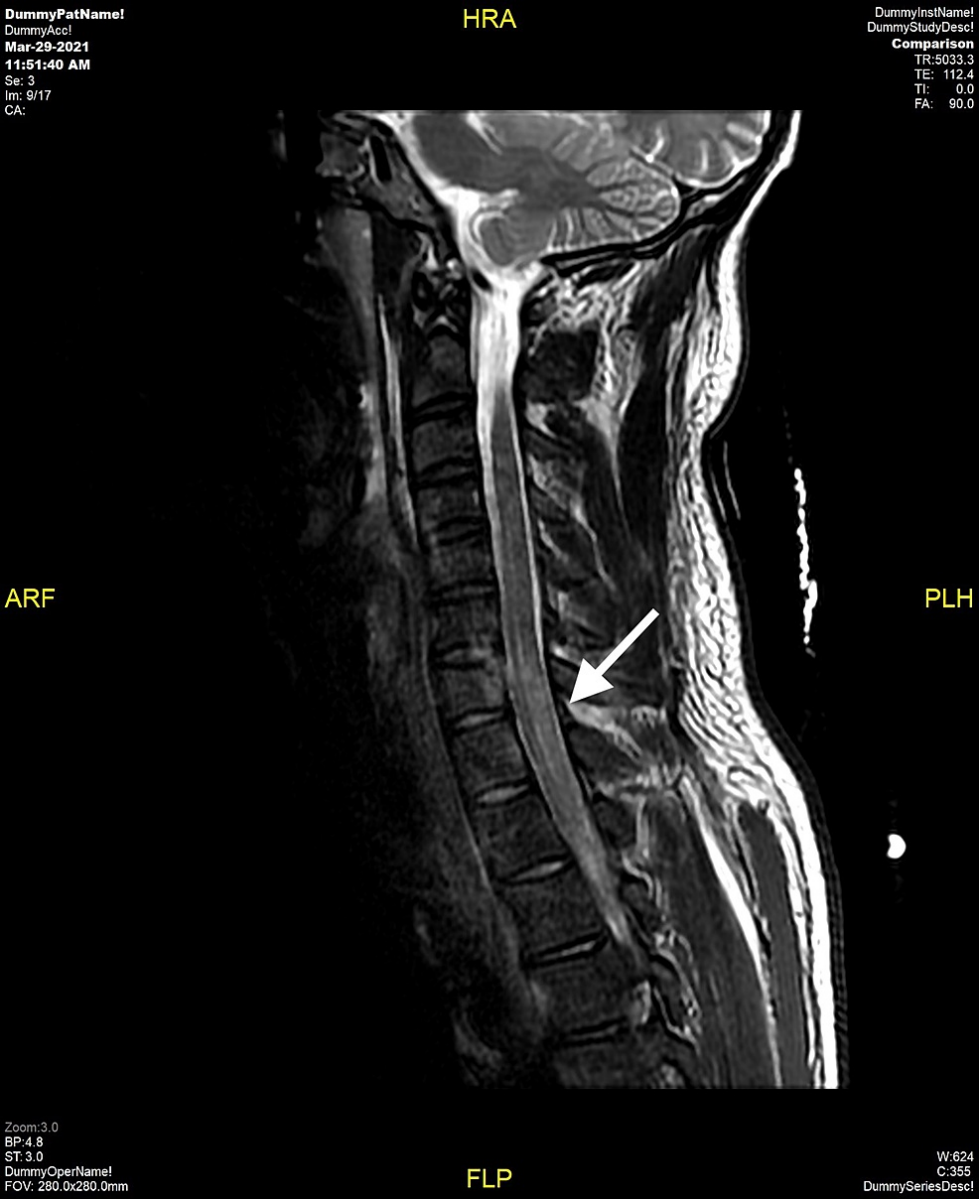
MRI of the Cervical Spine Showing Indistinct Edema from C5 to T1 (arrow)

Upon admission, full range of motion was documented in the upper and lower extremities. However, on hospital Day #1, lack of sensation was noted below T2, and the patient was unable to move his lower extremities. MRI of the cervical spine without contrast showed indistinct edema within the cervical cord beginning at the mid C5 level and extending down to the bottom of T1, suggesting a cord contusion secondary to blast injury (Figure 3).

Neurosurgery performed an emergent left C7-T1 foraminotomy and decompressive laminectomy of C6, C7, and T1, with fusion from C6-T1. The patient returned to the ICU with mechanical ventilation via a tracheostomy tube. Upon awakening, the patient was able to move his upper extremities but continued to lack sensation and movement in his lower extremities. Eventually, the patient was moved to the surgical floor. The patient continued to have gross movement of his upper extremities and had spontaneous movement of his lower extremities but still lacked sensation and controlled movement. The patient struggled with his mental health as he came to terms with his paraplegia. One month after his admission, the patient was discharged to a long-term acute care facility.

## Discussion

Self-inflicted gunshot wounds have increased significantly in incidence and are capable of devastating outcomes. Penetrating neck injuries are particularly deathly due to their high likelihood of damaging vital vascular organs like the carotid, subclavian, and vertebral arteries [2]. Similarly, pharyngoesophageal injuries carry high morbidity and mortality and are responsible for most delayed deaths from penetrating neck trauma [5]. Spinal cord injury from anterior penetrating neck trauma is a rare singular outcome. A retrospective analysis of a large level 1 trauma center found the incidence of cervical spine fractures to be 1.35 percent following gunshot wounds, thus supporting our conclusion that this is a rare outcome following penetrating neck trauma [6]. Additionally, all patients with cervical spine injury presented initially with neurological dysfunction, which varies with the presentation of our patient, who initially presented normally and developed progressively worsening neurological function. This progressive deterioration likely developed secondary to the cord contusion, which resulted from the shock wave produced by the bullet [7]. As penetrating neck injuries, particularly gun-shot wounds, become more prevalent in our communities, it is vital to be aware of all possible outcomes, as rapid recognition could prevent devastating outcomes.

## Conclusions

The complexity of injuries due to self-inflicted gunshot wounds to the neck poses many risks. The aim of this case report is to increase awareness of the rising incidence of self-inflicted gunshot wounds and the damage that can ensue. This patient presented with a gunshot wound that resulted in an unusual injury to the spinal cord. Traumas caused by gunshot wounds can be complicated by an unusual path and can be devastating depending on the extent of the injury. The majority of gunshot wounds occur in Zone 2, which further proves that each individual must have a treatment plan that is customized to decrease the morbidity, mortality, and long-term outcomes associated with self-inflicted gunshot wounds.

## References

[1] (2021). Gun violence archive. Gun Violence Archive, 8 Sept.

[2] Nowicki JL, Stew B, Ooi E (2018). Penetrating neck injuries: a guide to evaluation and management. Ann R Coll Surg Engl.

[3] Alao T, Waseem M (2021). Neck Trauma. https://www.ncbi.nlm.nih.gov/books/NBK470422/.

[4] Thal ER, Meyer DM (1992). Penetrating neck trauma. Curr Probl Surg.

[5] Asensio JA, Chahwan S, Forno W (2001). Penetrating esophageal injuries: multicenter study of the American Association for the Surgery of Trauma. J Trauma.

[6] Rhee P, Kuncir EJ, Johnson L (2006). Cervical spine injury is highly dependent on the mechanism of injury following blunt and penetrating assault. J Trauma.

[7] Patil R, Jaiswal G, Gupta TK (2015). Gunshot wound causing complete spinal cord injury without mechanical violation of spinal axis: case report with review of literature. J Craniovertebr Junction Spine.

